# Development of Three Different Anchovy-Based Fast-Food Products (Toast, Burger, and Pizza): Comparative Analysis of Sensory and Proximate Properties

**DOI:** 10.3390/foods14193329

**Published:** 2025-09-25

**Authors:** Fatma Delihasan Sonay, Barış Karslı, Emre Çağlak, Ayşe Kara, Özen Yusuf Öğretmen, Orhan Kobya

**Affiliations:** 1 Faculty of Fisheries, Recep Tayyip Erdogan University, 53100 Rize, Türkiye; baris.karsli@erdogan.edu.tr (B.K.); emre.caglak@erdogan.edu.tr (E.Ç.); ayse.kara1@erdogan.edu.tr (A.K.); ozenyusuf.ogretmen@erdogan.edu.tr (Ö.Y.Ö.); orhankobya@gmail.com (O.K.); 2 Department of Food and Nutritional Sciences, University of Reading, Reading RG6 6AP, UK

**Keywords:** anchovy, fast-food, healthy nutrition, nutritionally enriched food, seafood

## Abstract

This study aims to develop nutritionally improved alternative fast-food products by incorporating anchovy (*Engraulis encrasicolus*), a fish with high nutritional value, into three popular fast-food items (toast, burger, and pizza) frequently consumed by fast-food consumers. Anchovies, due to their rich content of omega-3 fatty acids, high-quality protein, vitamins A and D, and minerals, are a valuable food source for public health. Within the scope of this study, the nutritional compositions (crude protein, crude fat, crude ash, moisture, carbohydrate, energy) and sensory properties of the developed products were determined. According to the results of the analysis, the highest crude protein (18.64%) and crude ash (4.38%) content were found in anchovy-enriched toast, while the highest crude fat content (10.82%) was observed in anchovy burger (*p* < 0.05). Sensory analyses indicated that the panelists generally accepted all products. Specifically, the anchovy-enriched burger received the highest scores for appearance (90%) and aroma (40%). These findings demonstrate that anchovy-enriched fast-food products are both nutritionally rich and consumer-accepted, nutritionally improved food alternatives. Furthermore, this study identifies significant potential for utilizing aquatic products within the nutritionally enriched, seafood-based product sector.

## 1. Introduction

Food preferences and dietary behaviors worldwide are influenced by a variety of psychological, social, cultural, biological, and economic factors [1]. In particular, the transformation of economic structures, changes in lifestyles, and advancements in technology have significantly altered individuals’ nutritional habits. The fast pace of modern life, increasing urbanization, and enhanced opportunities for travel and communication have led people to prefer foods that are quick to prepare and easy to consume. Consequently, this shift has contributed to the global spread of fast food [2,3].

Despite their apparent appeal, stemming from ease of preparation and palatability, fast-food products are typically high in saturated fats, sugars, salt, and calories, while being low in essential nutrients. This nutritional imbalance has been associated with numerous health issues, including obesity, hypertension, diabetes, cardiovascular diseases, certain types of cancer, neurodegenerative disorders, autoimmune conditions, psychological problems, premature aging, and reduced physical performance [4]. It is evident that the complete elimination of fast-food consumption is an impractical objective. However, enhancing the nutritional value of these products and incorporating nutrient-dense additives emerge as pivotal strategies for promoting public health. At this point, seafood, as a valuable source of animal protein, merits particular attention. In the context of healthy dietary models, fish are of particular significance due to their high omega-3 fatty acid content, as well as their essential amino acids (threonine, valine, methionine, isoleucine, arginine, tryptophan, leucine, phenylalanine, lysine, and histidine), vitamins (A, D, and B), and minerals (sodium, potassium, calcium, magnesium, phosphorus, iron, copper, iodine, and fluoride). In view of the aforementioned properties, fish and fish-based products are categorized as “functional and valuable foods” [5,6,7]. Consuming fish has been shown to play a protective role in preventing cardiovascular disease, high cholesterol, and certain types of cancer. Additionally, maternal fish consumption before and during pregnancy is associated with improved neurodevelopment in infants and young children [8].

Anchovy (*Engraulis encrasicolus*) is a significant marine species due to its high biological value and rich nutritional profile. The latter is characterized by its high levels of omega-3 polyunsaturated fatty acids, high-quality protein, vitamins A and D, and a variety of essential minerals, especially phosphorus, sodium, and potassium. Moreover, the phospholipid components of anchovies have been demonstrated to exhibit immunomodulatory effects, thus offering potential benefits in the prevention of various diseases [9]. Anchovies are recognized not only in Turkey but also on a global scale as a prominent species due to their high catch volumes and biomass. Within the context of Turkey, which has an annual catch of approximately 153,175 tons [10], it occupies a strategic position in the fisheries sector, contributing to both ecological sustainability and economic development. Anchovies are a valuable resource for global food security and healthy nutrition strategies due to their rich nutrient composition and wide geographical distribution.

Anchovy, a fish of significant importance in Black Sea regional cuisine, appears in numerous traditional recipes, such as pan-fried anchovies, poached anchovies, anchovy and rice pilaf, anchovy patties, and anchovy omelet. In global cuisine, it features in dishes like “Pissaladière.” However, there are limited studies in the literature regarding the use of anchovies in fast-food products and their nutritional content.

The objective of this study was to evaluate anchovy, a fish that is abundantly harvested, has high nutritional quality, is cost-effective, and has not been widely utilized in diverse human food products, by incorporating it into three popular fast-food items (toast, burger, and pizza) frequently consumed by fast-food consumers. Furthermore, this study aimed to enhance the nutritional quality and value of these products, which are typically associated with low nutritional value and adverse health effects, by adding anchovies. To this end, the nutritional composition of the developed products was analyzed, and consumer sensory preferences were assessed.

## 2. Materials and Methods

### 2.1. Fish Materials

The European anchovies (*E. encrasicolus*) utilized in the present study were obtained from local fishermen. In order to maintain the cold chain, the anchovies were transported in insulated polystyrene containers filled with ice to the Processing Laboratory of the Recep Tayyip Erdoğan University Fisheries Research and Application Center. Upon arrival at the laboratory, the anchovies were meticulously gutted and fileted in preparation for processing. All other materials utilized in the study were obtained from local markets.

### 2.2. Preparation of Anchovy-Based Toast, Burger, and Pizza

**Figure 1:**
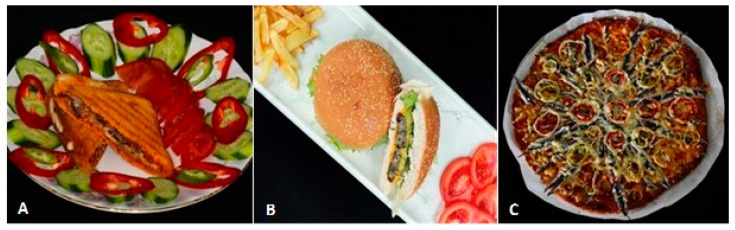
Photographic representation of anchovy-based toast (**A**), burger (**B**), and pizza (**C**) (original).

In this study, three distinct fast-food products were prepared using anchovy fish (Figure 1). To mitigate the characteristic odor of the fish, various spices and sauces were incorporated into the production of anchovy-based products, including anchovy toast, burgers, and pizza toppings. The products under discussion were prepared according to the recipes delineated in the work entitled “From Sea to Table: Anchovy” by Çağlak et al. [9]. The raw materials and production processes for the three different products are detailed below.

#### 2.2.1. Anchovy Toast

Initially, all the required ingredients for the sauce were homogeneously mixed. Prepared anchovy filets were cooked in a 28 cm diameter non-stick pan over high heat (surface temperature ≈ 170–180 °C) for approximately 3–4 min, until the internal temperature of the fish reached at least 72 °C and surface moisture had largely evaporated. A slice of toast bread was spread with one dessert spoon of the prepared sauce, followed by the placement of four anchovy filets on top. Sliced kashar cheese was then added over the filets, and an additional dessert spoon of sauce was spread on top. The sandwich was completed by placing another slice of toast bread on top and proceeded to the toasting process (Model TM 6206 G, Arçelik Inc., İstanbul, Türkiye). The toast was cooked in an electric toaster preheated to 200 °C for about 3–4 min per side, until both sides were golden brown and the internal temperature of the product reached 72 °C. This procedure was repeated until all ingredients were used, resulting in a total of 10 anchovy toasts (Figure 1A). The ingredients and their quantities used in preparing the anchovy toast are presented in Table 1.

#### 2.2.2. Anchovy Burger

Initially, anchovies, onions, garlic, and parsley were finely minced using a cleaver. To this mixture, eggs, additional parsley, allspice, black pepper, cumin, red pepper flakes, salt, baking soda, and breadcrumbs were added, and the mixture was kneaded until a homogeneous consistency was achieved. From this kneaded mixture, patties were formed to the size of hamburger buns. The prepared patties were cooked in a preheated non-stick pan with a thin layer of oil over medium-high heat (≈170–180 °C) for about 4–5 min per side, until both surfaces were evenly browned and the internal temperature reached at least 72 °C. The cooked patties were placed inside hamburger buns, and sliced tomatoes, lettuce leaves, and cheddar cheese were added to complete the anchovy burgers (Figure 1B). A total of 16 anchovy burgers were prepared using this method. The ingredients and their respective quantities used to prepare anchovy burgers are presented in Table 2.

#### 2.2.3. Anchovy Pizza

The production of pizza in this study comprised three primary stages: preparation of the tomato sauce, dough kneading and shaping, and baking. The detailed procedural steps are outlined below:

Tomato sauce preparation: The tomato sauce was prepared by first adding crushed garlic to liquid oil and lightly sautéing it. Subsequently, tomato paste was incorporated and sautéed for approximately 1–2 min. Following this, grated tomatoes, granulated sugar, and salt were added. After sautéing this mixture for about 1 min, water was introduced, and the mixture was boiled for 5 min. Upon removing from the heat, finely chopped basil and oregano were added, and the mixture was allowed to cool.

Pizza dough preparation: For the dough, flour, yeast, sugar, water, milk, cornmeal, liquid oil, and salt were combined in a bowl and kneaded for 5 min. The resulting dough was then covered and left to ferment for 30 min. After this period, the dough was divided into two equal portions, and each was rolled out thinly on a floured surface using a rolling pin.

Pizza formation and baking: A baking tray was lined with parchment paper and coated with a mixture of butter and tomato paste. The rolled-out dough was then placed on the tray, and its surface was pierced with a fork. The previously prepared tomato sauce was spread over the dough, followed by a layer of grated kashar cheese and the remaining pizza toppings. The prepared pizza was baked in a preheated electric oven (Model F 8440-1 B, Arçelik Inc., İstanbul, Türkiye) at 200 °C (top-and-bottom heating) for 15 min, then with the bottom element only for an additional 5 min, until the crust was golden brown and the internal temperature of the product reached at least 72 °C (Figure 1C). A total of 2 pizzas were prepared using this method. All ingredients and their quantities used in pizza preparation are detailed in Table 3.

### 2.3. Proximate Composition Analyses

The procedures recommended by the Association of Official Analytical Chemists (AOAC) were used to determine the crude protein, crude fat, crude ash, and moisture of the samples. For the proximate analyses, the food samples (hamburger, pizza, and toast) were first cooled at room temperature (22 ± 2 °C) for 10 min after cooking and then homogenized. All components of the product (e.g., bun, meat patty, tomato, and lettuce for the hamburger; dough, cheese, sauce, and toppings for pizza; bread, cheese, and filling for toast) were combined to obtain representative mixtures. Approximately 5 g of homogenized sample was used for each analysis. The crude protein content of the samples was determined using the Kjeldahl method (Method 978.04) [11,12]. In this method, the nitrogen content of the samples was measured, and the obtained value was multiplied by a factor of 6.25 to calculate the protein content. The crude fat content was determined using the Soxhlet extraction method (Method 930.09) with petroleum ether as the extraction solvent [11,12]. The moisture content of the samples was ascertained by the standard drying method (Method 985.14) [13]. Samples were dried in a hot-air oven at 105 °C until they reached a constant weight. Crude ash content was determined by incinerating the samples in a muffle furnace at 550 °C (Method 930.05) [11,12]. Blanks were run in parallel to check for background contamination, and all measurements followed AOAC-recommended quality control practices to ensure accuracy and reproducibility.

The proximate composition of samples, including crude protein (1), crude fat (2), moisture (3), crude ash (4), carbohydrates (5), and energy (6) were calculated using the following formulas [14,15,16]:

Crude protein (%) = (V × 0.14 × 6.25)/(W)(1)

where V is the titration volume (mL) of 0.1 N H_2_SO_4_ used, and W is the weight of the sample (g).

Crude fat (%) = (Weight of fat/Weight of sample) × 100(2)

Moisture (%) = ((Initial weight − Dry weight)/Initial) × 100(3)

Crude ash (%) = (Ash residue mass/Sample mass) × 100(4)

Carbohydrate (%) = 100 − (Crude protein + Crude fat + Crude ash + Moisture)(5)

Energy (kcal/100 g) = (Crude protein × 4) + (Crude fat × 9) + (Carbohydrate × 4)(6)

### 2.4. Sensory Evaluation

**Figure A1:**
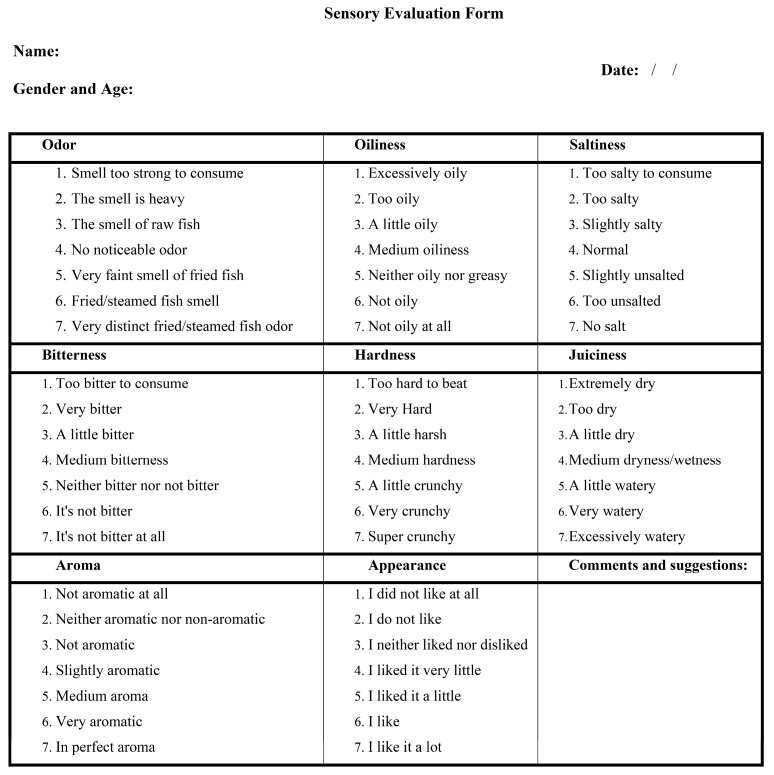
Sensory evaluation form of anchovy-based toast, burger, and pizza.

The sensory evaluation was conducted using a descriptive analysis performed by a trained panel of 20 members, consisting of researchers from the Faculty of Fisheries, Recep Tayyip Erdoğan University, Türkiye. The panelists ranged in age from 27 to 51, with a gender distribution of 55% male and 45% female. All provided verbal consent prior to participation. To ensure reliability of the assessments, the panelists attended training sessions to become familiar with the sensory attribute scales and definitions, and to calibrate their evaluations using reference samples. Participation was entirely voluntary. Test samples were portioned as follows: toast (≈100 g), burger (≈190 g), and pizza (served as slices of ≈100 g). All products were presented on plates of identical color and shape to minimize visual bias, and water was provided between samples to cleanse the palate. And to minimize external factors and ensure consistency, the sensory evaluations were conducted under standardized conditions (lighting, temperature, and ventilation). The selection of sensory attributes was based on a review of previous studies on fish-containing products and fast-food formulations, and further refined during preliminary training sessions with the panelists to ensure their relevance and clarity for anchovy-based products. Panelists evaluated the products based on the following criteria: odor, oiliness, saltiness, bitterness, juiciness, aroma, and appearance. For the sensory analysis, the evaluation form with predefined categorical descriptors was prepared by revising from previous studies (Figure A1) [14].

### 2.5. Statistical Analysis

The proximate composition results of anchovy toast, burger, and pizza products were statistically compared using a one-way analysis of variance (ANOVA) at a 95% significance level (*p* < 0.05). Tukey’s post hoc test was used to determine differences between groups. Data analysis was performed using Sigmaplot 11 (SYSTAT Software, Inc., Chicago, IL, USA) and Microsoft Office Excel 2016 Pro. Software.

## 3. Results and Discussion

The developed anchovy-based toast, burger, and pizza products were then evaluated in terms of proximate composition and sensory characteristics, as described in the following sections.

### 3.1. Proximate Composition of Anchovy Toast, Burger, and Pizza

In this study, the chemical compositions of three distinct products developed using anchovies (anchovy toast, anchovy burger, and anchovy pizza) were evaluated and compared with those of fresh anchovies. As presented in Table 4, statistically significant differences were observed among the products in terms of crude protein, crude fat, crude ash, moisture, carbohydrate, and energy content (*p* < 0.05). These three anchovy-based products were then compared with products containing different fish species. While anchovy burger and anchovy pizza were evaluated against products made with various fish types and different processing methods, no direct research precedent was found for anchovy toast. Therefore, anchovy toast was compared with sandwich products made from salmon and tuna (Table 5).

As anticipated, the highest moisture content (%) was observed in fresh anchovies. Conversely, a significant decrease in moisture content was observed in heat-treated products, with this difference being statistically significant (*p* < 0.05). Among the processed products, anchovy pizza exhibited a moisture content of 56.14%, followed by anchovy burger at 48.25%, and anchovy toast at 38.83%. Moisture content is inversely proportional to the relative content of energy, protein, and lipids; a decrease in moisture leads to an increase in lipid and protein content, thereby increasing the energy density of fish products [17]. Furthermore, this reduction in moisture can be considered a potential advantage, as it may enhance microbiological stability and extend the shelf life of the products [17]. The moisture content of the products varied depending on the different fish species and cooking techniques used (Table 5).

The highest crude protein (%) content was found in anchovy toast, crude fat (%) in anchovy burger, crude ash (%) in anchovy toast, and carbohydrate (%) in both anchovy toast and burger (*p* < 0.05). These results differed significantly from the proximate values of fresh anchovies (Table 4) (*p* < 0.05). These discrepancies can be attributed to the proportion of auxiliary/additive ingredients used (including protein, fat, carbohydrate, mineral, and vitamin values), as well as processing methods and cooking techniques. The energy value of the processed products increased significantly compared to fresh anchovies. Anchovy toast recorded the highest energy content at 266 kcal/100 g, followed by the burger at 251 kcal/100 g and pizza at 209 kcal/100 g. Fresh anchovy, in contrast, provides only 111 kcal/100 g. This increase can be explained by both moisture loss and the contribution of added ingredients (fat and carbohydrates) to the product. Furthermore, these values can vary due to factors such as the fishing season, physiological structure of the fish, environmental changes, contributions from other ingredients added during preparation, and cooking techniques. Consequently, as shown in Table 4, the nutritional values of anchovy-based processed products vary depending on their formulation and the cooking method used. Anchovy toast stands out with its high protein, ash, and energy content, while the anchovy burger offers a richer profile in terms of fat and carbohydrates. Anchovy pizza, on the other hand, provides comparatively lower calorie. It is believed that these products can offer consumers both nutritious and practical alternatives to traditional anchovy consumption. Proximate values for toast, burger, and pizza made with different fish species are presented in Table 5.

From a consumer health perspective, replacing commonly consumed fast-food products with more sustainable seafood-based alternatives is considered a promising approach to improve nutritional profiles. With the growing awareness of health problems associated with high meat consumption, the meat industry has sought to enhance the public perception of meat-based functional foods by substituting certain natural saturated animal fats with healthier unsaturated fats derived from alternative sources ingredients. In this context, the beneficial components of fish and other seafood play a crucial role. In the present study, the proximate composition values (Table 5) reveal notable differences compared to those of conventional meat-based products, highlighting the nutritional advantages of incorporating seafood [18]. The proximate composition of the anchovy burger developed in this study (Table 4) revealed substantial nutritional differences when compared with conventional fast-food burgers reported in the literature (Table 5). In the present study, proximate analyses were performed on the entire product, including the bun, patty, and other ingredients, whereas some previous studies focused solely on the burger patty. This methodological difference may explain the variations observed when comparing our results with other anchovy burgers (protein: 18.49%, fat: 4.40%, carbohydrate: 3.96%, and energy: 125.5 kcal/100 g), commercial fast-food products (protein: 12.9–14.8%, fat: 10.1–10.6%, carbohydrate: 26.8–30.3%, and energy: 261–264 kcal/100 g), and 100% beef burgers (protein: 20.4%, fat: 22.1%, carbohydrate: 0.88%, and energy: 292 kcal/100 g) [19].

When evaluating toast, the nutritional composition varies depending on the ingredients added to the bread, with reported values ranging from 9.16% to 35.97% protein, 9.49% to 33.40% fat, 27.08% to 75.04% carbohydrate, and 252 to 751 kcal/100 g [20]. The results of the present study show both similarities and differences compared to these reported values. Generally, the addition of processed products such as sucuk (Turkish sausage), sausage, and salami to toast may pose potential health risks due to their high fat, sodium, and preservative content.

Pizza is a versatile food product that can include a wide range of ingredients, from fruits and vegetables to red meat and poultry, and its flavor can be further enhanced with various sauces and spices. The nutritional composition of pizza varies depending on the specific ingredients used. According to the U.S. Department of Agriculture data for meat-topped pizzas, although the proximate values (protein: 19.19%, fat: 24.52%, carbohydrates: 5.65%, and energy: 321 kcal/100 g) [21] of anchovy-based pizzas are relatively lower compared to conventional meat-topped pizzas, the inclusion of anchovies enhances the overall healthfulness of the product.

The nutritional value of fast-food products varies considerably depending on the ingredients used. In particular, red meat and processed meat products have been associated with health problems such as cardiovascular diseases and metabolic disorders due to their high saturated fat and sodium content. In contrast, seafood, especially fish, holds an indispensable place in human nutrition due to its unique and highly optimized nutritional composition. Their fundamental value lies not only in providing highly bioavailable protein but also in serving as the primary source of long-chain polyunsaturated fatty acids, such as eicosapentaenoic acid (EPA) and docosahexaenoic acid (DHA), which are rarely found in terrestrial foods. Small pelagic fish, such as anchovies, are particularly advantageous due to their low mercury content and environmental sustainability [22,23,24,25]. From a nutritional functionality perspective, the contribution of fish extends far beyond their role as a source of energy or protein, and the incorporation of seafood into fast-food products enhances their nutritional value, offering healthier alternatives.

**Table 5:** Proximate composition of different fish-based toast, burgers, and pizzas.

Fish Species	Crude Protein (%)	Crude Fat (%)	Crude Ash (%)	Moisture (%)	Carbohydrate (%)	Energy (kcal/100 g)	References
**Fish toast/Sandwich**							
Salmon (smoked)	9.82	5.00	-	-	34.75	82	[26]
Tuna	12.11	4.57	-	-	23.17	183	[27]
**Fish burger**							
European anchovy (*Engraulis encrasicolus*)	18.49 ± 0.74	4.40 ± 0.22	1.31 ± 0.04	66.06 ± 2.34	3.96 ± 0.11	125.5 ± 2.34	[19]
Pacu (*Pyaractus brachypomus*)	15.27 ± 0.03	17.60 ± 0.06	1.64 ± 0.03	61.04 ± 0.01	6.08 ± 0.02	-	[28]
Boquichico (*Prochilodus nigricans*)	17.54 ± 0.04	16.43 ± 0.09	1.54 ± 0.08	61.81 ± 0.07	4.25 ± 0.20	-	
Bujurqui (*Chaetobranchus flavescens*)	17.77 ± 0.02	15.48 ± 0.08	1.60 ± 0.06	65.36 ± 0.16	1.39 ± 0.08	-	
Catfish (*Clarias gariepenius*)	8.15 ± 0.90	11.79 ± 0.19	1.03 ± 0.07	47.15 ± 1.60	31.36 ± 0.86	-	[17]
Mackerel (*Scomber scombrus*)	6.02 ± 0.98	14.57 ± 1.47	1.19 ± 0.11	44.61 ± 0.39	33.05 ± 1.96	-	
Atlantic croaker (*Micropogonias undulates*)	5.92 ± 1.28	15.58 ± 0.62	1.28 ± 0.08	41.88 ± 2.08	34.78 ± 0.62	-	
European hake (*Merluccius merluccius*)	5.59 ± 0.51	11.34 ± 0.69	1.35 ± 0.05	51.76 ± 0.76	29.38 ± 0.62	-	
Common carp (*Cyprinus carpio*)	18.12 ± 0.01	13.24 ± 0.06	2.69 ± 0.01	55.25 ± 0.08	-	-	[29]
Tilapia (*Oreochromis niloticus*)	16.55 ± 0.12	6.57 ± 0.41	2.69 ± 0.09	68.28 ± 0.33	-	-	[30]
Grass carp (*Ctenopharyngodon idella*)	16.42 ± 0.57	6.64 ± 0.15	2.98 ± 0.09	69.46 ± 0.89	-	-	[31]
Rainbow trout (*Oncorhynchius mykiss*)	17.50 ± 0.25	2.87 ± 0.10	3.33 ± 0.30	61.87 ± 0.38	14.56 ± 0.30		[32]
**Fish pizza**							
Tilapia and Salmon	17.00 ± 0.51	6.64 ± 0.94	5.33 ± 0.47	40.10 ± 0.47	30.94 ± 1.62	251.46 ± 2.50	[33]
Carp (*Cyprinus carpio*)	15.34 ± 0.18	4.31 ± 0.03	0.93 ± 0.02	-	79.42 ± 0.22	-	[34]
Tuna (*Tunnus* spp.)	18.94 ± 0.24	6.10 ± 0.41	3.54 ± 0.28	26.54 ± 1.11	44.87 ± 0.84	310.20 ± 3.89	[35]
Argentine anchovy (*Engraulis anchoita*)	11.9 ± 0.88	14.2 ± 0.66	3.41 ± 0.03	51.01 ± 0.07	19.05 ± 0.16	251.6	[36]

### 3.2. Sensory Evaluation

**Figure 2:**
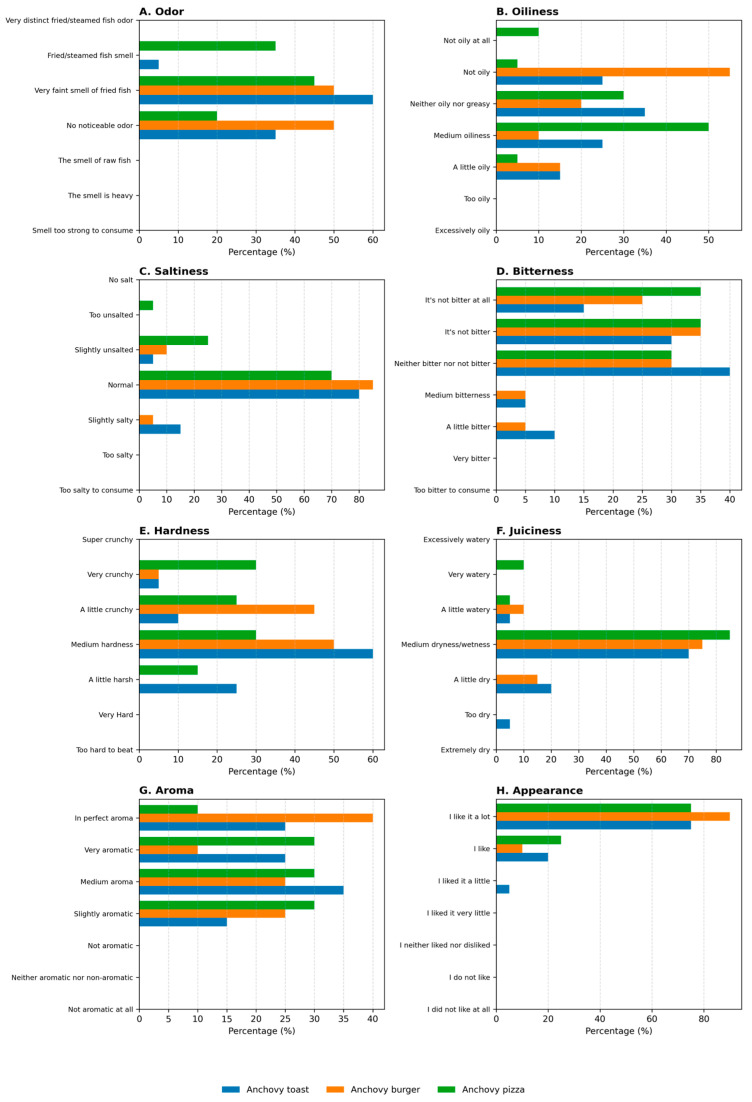
Sensory scores of anchovy toast, burger, and pizza products (*n* = 20).

The evaluation of sensory attributes is a fundamental criterion in determining consumer preferences for food products. It is a widely used analytical method for revealing changes in qualities such as texture, taste, flavor, and aroma [8,37,38]. As with other food categories, sensory analysis plays a crucial role in the quality control processes of seafood products, serving as a significant determinant of consumer acceptance [39,40]. In this study, three different fast-food products (anchovy toast, anchovy burger, and anchovy pizza) were developed using anchovies to create nutritionally enriched options. Their sensory characteristics were evaluated, and the results of the sensory analysis are presented in Figure 2.

According to the panelists’ evaluations, 60% of the anchovy toast samples were classified as having a “very faint smell of fried fish.” For anchovy burger samples, 50% of panelists reported “no noticeable odor,” while the other 50% noted a “very faint smell of fried fish.” In the case of anchovy pizza samples, 45% of panelists evaluated them as having a “very faint smell of fried fish” (Figure 2A). Notably, no negative odor perceptions such as “smell too strong to consume,” “the smell is heavy,” “the smell of raw fish,” or “very distinct fried/steamed fish odor” were reported by panelists for any of the three products. These findings suggest the effectiveness of techniques employed during product preparation, including the controlled reduction in anchovy moisture and the incorporation of sauces and spices. Since fish odor is a significant factor influencing fish consumption, the successful suppression of this odor through these techniques likely contributed to an increased preference for consuming fish.

Regarding the oiliness criterion, anchovy toast was rated as “neither oily nor greasy” by 35% of panelists. In comparison, the anchovy burger was categorized as “not oily” by 55%, and the anchovy pizza as “medium oiliness” by 50% (Figure 2B). Although proximate analysis revealed that the burger had the highest fat content (10.82%), this was not perceived as oiliness by the panelists. This apparent discrepancy may be explained by the integration of fat within the patty and cheese matrix, the absorption of fat into the bread and vegetables, and the juiciness released during mastication, all of which reduce the perception of free surface oil. Thus, the sensory outcome reflects perceived oiliness rather than absolute fat percentage. These findings indicate that anchovy toast and pizza have a more balanced fat content, whereas the anchovy burger stands out as a less oily product. The saltiness level was evaluated as normal for all three products, suggesting that the amount of salt and spices used was appropriate (Figure 2C). In terms of bitterness, none of the products were deemed “very bitter” or “too bitter to consume,” indicating broad consumer acceptability and that the bitterness was not excessive. For anchovy toast, 40% of panelists selected “neither bitter nor not bitter,” while for anchovy burger, 35% chose “it’s not bitter.” For anchovy pizza, 35% of panelists opted for “it’s not bitter” and “it’s not bitter at all” (Figure 2D). These results suggest that the taste profile of the products is generally neutral, with no pronounced bitterness.

Panelists evaluated all three products as having “medium hardness,” with anchovy toast, anchovy burger, and anchovy pizza receiving ratings of 60%, 50%, and 30% in this category, respectively (Figure 2E). This indicates that the products have an acceptable texture and a structure that allows consumers to chew them comfortably. In the “medium dryness” category, anchovy toast, anchovy burger, and anchovy pizza were rated at 70%, 75%, and 85%, respectively (Figure 2F). This result suggests that the pizza was particularly drier, while the toast and burger had a moister consistency. The sauces and spices used in the preparation of the products significantly influenced their aroma profiles, as indicated by the non-selection of options such as “not aromatic at all,” “neither aromatic nor non-aromatic,” and “not aromatic,” demonstrating that the products offered a rich aroma profile (Figure 2G). Specifically, the anchovy burger was chosen by 40% of the panelists as having “in perfect aroma,” revealing that its aromatic components were well-received and satisfactory.

In appearance evaluations, all products received high levels of approval; the panelists’ preference for the “I like it a lot” option was recorded at 75% for anchovy toast and pizza, and 90% for anchovy burger (Figure 2H). These results indicate that the visual presentation of all three developed products generally met consumer expectations. The anchovy burger’s highest approval rating may be attributed to its formal integrity, familiarity, and the balanced appearance of its components. It is foreseen that the burger form’s consistency with traditional fast-food perception and its regular, compact structure might have contributed to its greater visual appeal.

## 4. Future Research

The innovative fish-based products, such as anchovy toast, anchovy burger, and anchovy pizza, hold significant potential for evaluation within academic research and the food industry. Given that anchovies are naturally rich in omega-3 fatty acids, protein, calcium, and various microelements, these products offer substantial advantages for positioning them within the functional food category.

Future systematic investigations into these products should encompass variables like consumer perception, packaging design, brand positioning, and cooking methods. Such research would contribute to health-oriented dietary models and promote broader fish consumption. Furthermore, to optimize the industrial production processes of these products, we recommend developing frozen forms that consider parameters such as freezing conditions, pre-cooking steps, and sauce content. For shelf-life determination, it is crucial to meticulously analyze factors during storage, including temperature, packaging type, and protection against light and moisture. This analysis should involve detailed assessments of lipid oxidation, microbial growth, chemical deterioration, and changes in texture and color. In addition, key areas for future studies also include cost-effectiveness analyses of these products, their integration with various cooking and presentation techniques, and a comparative evaluation of gastronomic innovation and consumer acceptance levels.

## 5. Conclusions

This study aimed to enhance the nutritional value and evaluate the sensory properties of three globally popular fast-food products (toast, burger, and pizza) by incorporating anchovies, yielding positive results. These anchovy-enriched products represent an innovative and nutritionally enriched approach to product development, merging local dietary habits with global fast-food culture. The findings indicate that these products are not only nutritionally beneficial but also sensorially acceptable to consumers. The escalating global population and fast-paced lifestyles increasingly steer individuals towards unhealthy dietary habits, fostering severe health issues such as obesity. In this context, re-establishing healthier and more balanced eating habits is of paramount importance. The anchovy-based products developed in this study offer an alternative solution to this need, thanks to their high nutritional value and convenience for practical consumption. Consequently, these types of products can significantly contribute to increasing the consumption of aquatic products. They hold substantial potential for supporting the “Slow Food” movement and promoting the development of alternatives with traditional and nutritionally valuable ingredients, replacing processed products of low nutritional value. Future studies can provide a more comprehensive evaluation of the industrial production, marketability, and consumer acceptance of these products.

## Figures and Tables

**Table 1:** Ingredients and quantities used in the preparation of anchovy toast (10 pieces).

For Anchovy Toast		For Sauce	
Ingredients	Quantity	Ingredients	Quantity
Anchovy (fileted) Kashar cheese Toast bread Butter	300 g 200 g 1 pack 2 tablespoons (≈50 g)	Pepper paste Tomato paste Olive oil Breadcrumbs Garlic Red pepper flakes Black pepper Thyme Mint Cumin Red pepper powder Salt	2 tablespoons (≈60 g) 2 tablespoons (≈60 g) Half a glass (≈100 mL) 1 tablespoon (≈10 g) 1 clove (crushed) 1 teaspoon (≈2 g) 1 teaspoon (≈2 g) 1 teaspoon (≈1 g) 1 teaspoon (≈0.5 g) 1 teaspoon (≈2 g) 1 teaspoon (≈2 g) 1 teaspoon (≈4 g)

**Table 2:** Ingredients and quantities used in anchovy burger preparation (16 pieces).

For Anchovy Burger		For Anchovy Meatballs	
Ingredients	Quantity	Ingredients	Quantity
Large hamburger buns Sliced cheddar cheese Lettuce Tomatoes (cut into rings)	2 packages 1 package A few leaves 2 large	Anchovy (fileted) Onions Garlic Allspice Black pepper Cumin Red pepper flakes Salt Egg Baking soda Parsley Breadcrumbs Oil (to fry)	500 g 2 medium 3 cloves (crushed) 2 teaspoons (≈4 g) 2 teaspoons (≈4 g) 1 teaspoon (≈2 g) 2 teaspoons (≈4 g) 3 teaspoons (≈12 g) 2 pieces 2 teaspoons (≈8 g) Half a bunch 1.5 tablespoons (≈15 g) 1 cup (≈200 mL)

**Table 3:** Ingredients and quantities used in the preparation of anchovy pizza (2 pieces).

**For Dough**		**For Sauce**	
**Ingredients**	**Quantity**	**Ingredients**	**Quantity**
Flour Yeast Sugar Warm water Warm milk Corn flour Oil Salt	3 cups (≈360 g) 1 pack instant (10 g) 1 tablespoon (≈16 g) Half a cup (≈100 mL) Half a cup (≈100 mL) Half a teacup (≈30 g) Half a teacup (≈50 mL) 1 teaspoon (≈4 g)	Sugar Oil Garlic Tomato Tomato paste Fresh basil * Thyme Salt Water	1 teaspoon (≈3 g) 3 tablespoons (≈30 mL) 1 large clove (crushed) 1 large 1 tablespoon (≈30 g) 5 leaves 1 teaspoon (≈1 g) Half a teaspoon (≈2 g) Half a glass (≈100 mL)
**On Pizza**		**For the Base of the Baking Tray**	
**Ingredients**	**Quantity**	**Ingredients**	**Quantity**
Anchovy (fileted) Grated kashar cheese Capia peppers Village peppers Charleston peppers	400 g 300 g 2 pieces 2 pieces 2 pieces	Butter Pepper paste	1 tablespoon (≈25 g) 1 tablespoon (≈30 g)

* If you do not have any, use half a teaspoon of dried basil.

**Table 4:** Proximate composition of anchovy toast, burger, and pizza.

Products	Crude Protein (%)	Crude Fat (%)	Crude Ash (%)	Moisture (%)	Carbohydrate (%)	Energy (kcal/100 g)
Anchovy Toast	18.64 ± 0.84 ^a^	7.85 ± 0.24 ^bc^	4.38 ± 0.07 ^a^	38.83 ± 0.33 ^d^	30.30 ± 0.21 ^a^	266
Anchovy Burger	9.89 ± 0.78 ^d^	10.82 ± 0.11 ^a^	2.46 ± 0.20 ^b^	48.25 ± 0.05 ^c^	28.58 ± 0.80 ^a^	251
Anchovy Pizza	12.75 ± 0.21 ^c^	8.32 ± 0.09 ^b^	2.01 ± 0.02 ^c^	56.14 ± 0.15 ^b^	20.78 ± 0.49 ^b^	209
Raw anchovy	16.71 ± 0.24 ^b^	4.50 ± 0.17 ^c^	1.41 ± 0.03 ^d^	76.45 ± 0.21 ^a^	0.93 ± 0.17 ^c^	111

Values are means ± SD of duplicate determinations. Values in the same column with different superscripts were significantly (*p* < 0.05) different.

## Data Availability

The raw data supporting the conclusions of this article will be made available by the authors on request.
